# Liposarcoma of the Spermatic Cord Masquerading as an Inguinal Hernia

**DOI:** 10.1155/2014/735380

**Published:** 2014-01-30

**Authors:** William Londeree, Tamie Kerns

**Affiliations:** ^1^Department of Medicine, Tripler Army Medical Center, 1 Jarrett White Road, Honolulu, HI 96859, USA; ^2^Hematology and Oncology Service, Tripler Army Medical Center, Fort Washington, PA 19034, USA

## Abstract

This is a case of a 70-year-old male who presented with a mass in his right testicle. He was treated with antibiotics for epididymitis while undergoing serial ultrasounds for one year due to testicular swelling and pain. His fourth ultrasound revealed a mild hydrocele with a large paratesticular mass of undescribed size, superior to the right testicle, thought to be an inguinal hernia. Preoperative CT scan demonstrated a large fat-containing inguinal hernia extending into the scrotal sac. An inguinal hernia repair was complicated by fatty tissue surrounding the testicle requiring a right orchiectomy. Pathology review of the tissue demonstrated well-differentiated liposarcoma with a small focus of dedifferentiation grade 2 tumor. Tumor was identified at the inked margins indicating an incomplete resection. It was decided that no further surgical intervention was needed and the patient would undergo surveillance for local tumor recurrence. Six-month follow-up MRI scan was negative for any recurrence of disease. A liposarcoma presenting as a paratesticular mass with spermatic cord involvement is rare, and imaging studies may fail to distinguish a liposarcoma from normal adipose tissue.

## 1. Introduction

Liposarcoma is a malignant soft tissue sarcoma typically occurring in the thigh or retroperitoneum in an adult. However, a liposarcoma presenting as a paratesticular mass with spermatic cord involvement is rare. The first reported case was in 1845 and only 100 cases are found in the literature [1]. The diagnosis is hard to obtain due to the tumor being low grade and difficult to distinguish from an inguinal hernia due to adipose tissue composition when viewed on MRI, CT, or ultrasound [2].

## 2. Case

A 70-year-old male presented to his internal medicine primary care physician with a mass and swelling of his right testicle. Initial evaluation demonstrated a negative polymerase chain reaction (PCR) test for gonorrhea and chlamydia. Beta-human chorionic gonadotropin (HCG), lactate dehydrogenase (LDH), and *α*-fetoprotein (AFP) tests were within normal limits. He was treated empirically with antibiotics for epididymitis before the laboratory results returned. A screening testicular ultrasound demonstrated an extratesticular mass thought to be an inguinal hernia because of its reducibility on exam. Since it was reducible and he was not in pain, no further workup or surgical intervention was performed. On continued follow-up appointments with his primary care physician, he noted increasing swelling and pain at the site of the mass, prompting a repeat ultrasound. The ultrasound revealed a mild hydrocele with a large paratesticular mass of undescribed size superior to the right testicle, which was still believed to be consistent with an inguinal hernia.

**Figure 1:**
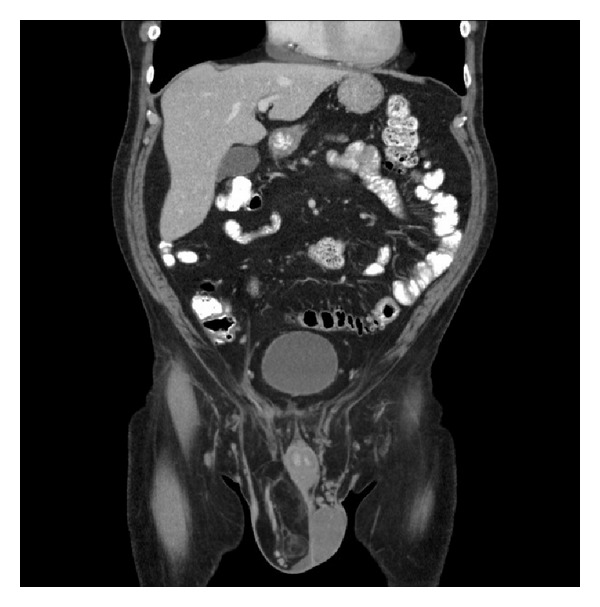
CT abdomen/pelvis read as a large fat-containing inguinal hernia present on the right side extending into the scrotal sac. No bowel loops are contained but there is a significant amount of omentum and fat present.

**Figure 2:**
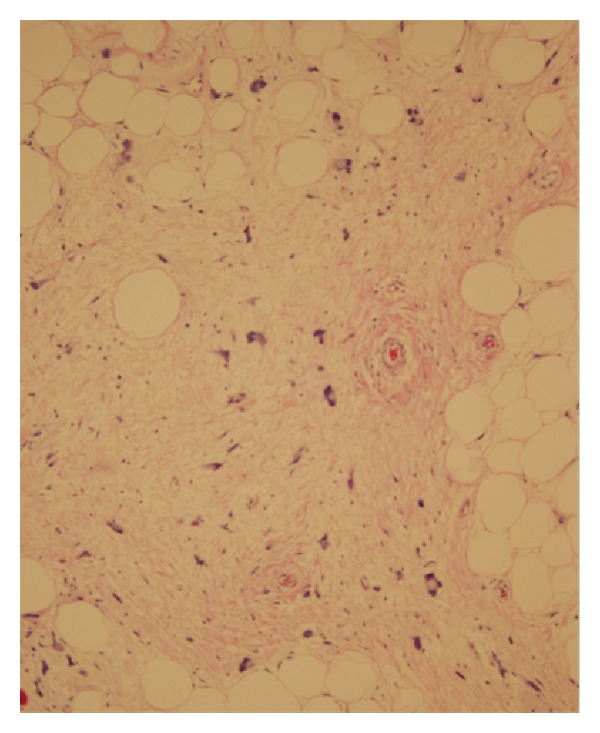
Light microscopy displays expanded hypercellular stroma with atypical adipocytes with enlarged nuclei and hyperchromasia.

**Figure 3:**
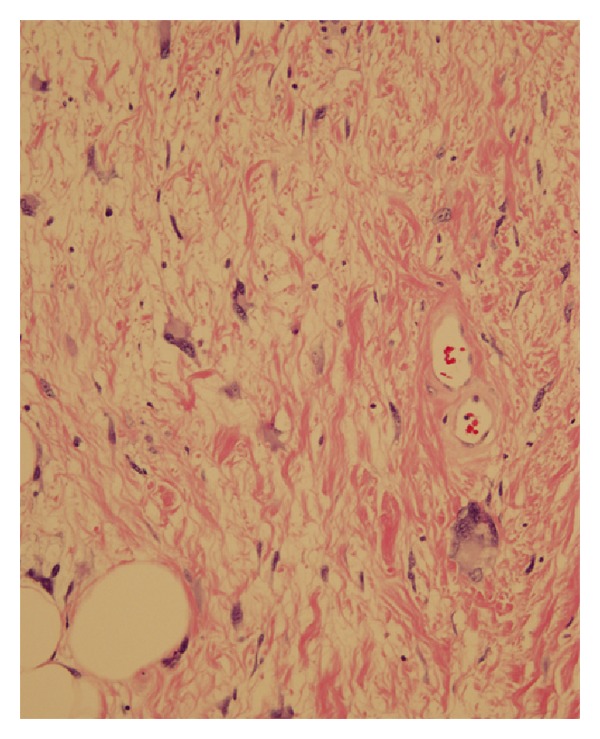
Light microscopy demonstrates atypical adipocytes with enlarged nuclei, hyperchromasia, and intranuclear vacuoles.

Due to the progressive testicular pain, general surgery was consulted for a right sided inguinal hernia. A preoperative CT scan demonstrated a large fat-containing inguinal hernia on the right side extending into the scrotal sac (Figure 1). On physical exam the mass seemed to be reducible but was very tender to palpation. A urology consult was not obtained prior to surgery since the mass appeared to be composed of adipose tissue and was believed to be an inguinal hernia. He underwent surgery for an inguinal hernia repair, and a prolene hernia system mesh was placed in the preperitoneal space covering the entire myopectineal orifice for a small indirect inguinal hernia. Mobilization of the distal cord and testes was complicated by fatty tissue surrounding the spermatic cord and right testicle. Urology was consulted intraoperatively and a right sided radical orchiectomy was performed; a spermatic cord mass of 14 × 8 × 4 cm which enveloped the spermatic cord was resected with 12 cm of the spermatic cord. The pathology was sent to Joint Pathology Center (JPC), Bethesda, MD. The tissue was consistent with a well-differentiated liposarcoma with a small focus of dedifferentiation grade 2/4 tumor (Figures 2 and 3). Tumor identified at the inked margins indicated incomplete resection. A six-month postoperative CT scan and MRI were negative for any recurrence of disease. The case was discussed in a multidisciplinary tumor board and with the patient. The patient was given treatment options of further surgical resection or surveillance. Given his age, the patient chose surveillance because he did not desire further surgical intervention. The board agreed it was reasonable to conduct surveillance screening due to the anatomical location and the tumor being well differentiated with only a small amount of dedifferentiated tumor present. The patient had serial surveillance with MRI at one year. At eighteen months after resection, there has been no recurrence of disease.

## 3. Discussion

The initial workup for a testicular mass should reflect the potential etiologies in the differential, which include an inguinal hernia, hydrocele, varicocele, epididymitis, orchitis, epidermoid cyst, germ cell tumors, lymphoma, metastases, and lymphedema [3]. The evaluation should exclude malignancy.

Germ cell tumors normally present as a painless mass and can be evaluated with ultrasound and serum levels of *α*-fetoprotein (AFP), human chorionic gonadotropin (hCG), and lactate dehydrogenase (LDH). A chest X-ray can be ordered if there is a high suspicion for metastatic disease [4]. When the ultrasound, tumor markers, urinalysis, and gonorrhea/chlamydia screening are negative, further investigation of the mass should be conducted with concern for lymphoma. The gold standard in the staging of soft tissue tumors is the MRI. In this case, a CT was performed instead, as the mass was believed to be an inguinal hernia preoperatively. It demonstrated a large fat collection consistent with an inguinal hernia although actual tissue pathology revealed a well-differentiated liposarcoma with a small focus of dedifferentiation grade 2/4 tumor.

Liposarcoma is the most common type of soft tissue sarcoma. It can be divided into five histological subtypes from highest to lowest incidence: well-differentiated, dedifferentiated, myxoid, round cell, and pleomorphic. The most common of these are the well-differentiated tumors, which are low grade tumors, followed by dedifferentiated tumors, which are higher grade tumors [2]. A patient typically presents with pain due to compression or invasion of the anatomical structures in the thigh or retroperitoneum [5].

Correctly diagnosing a liposarcoma of the spermatic cord is difficult due to the anatomical location in which it presents and the appearance of the mass as fatty tissue on imaging studies [1, 2]. Unlike germ cell tumors, ultrasound can have difficulty in distinguishing well-differentiated liposarcomas from adipose tissue. MRI with gadolinium enhancement is the optimal imaging choice because it has demonstrated ability in extremities to better identify viable tumor tissue versus surrounding reactive tissues [2, 6]. In comparison, CT or MRI can be used with equal utility for retroperitoneal soft tissue sarcomas [7–9]. However, a liposarcoma of the spermatic cord may be mistaken as a hernia due to its fatty composition with MRI or CT demonstrating a fatty mass, especially in well-differentiated tumor [2, 10]. More dedifferentiated histological tumors may lose their adipose appearance and can lead to a preoperative diagnosis. Two case reports have described a preoperative diagnosis made with US and MRI; however, histology from both cases revealed a myxoid liposarcoma which is a more dedifferentiated tumor than the one presented in the case [11, 12]. Given its location in the spermatic cord, there is no contrasting anatomy, such as muscle, that would indicate that the tumor is invasive. Since invasion is not seen and only a fatty mass is visualized, the mass appears to be inguinal hernia which leads to a delay in treatment [2].

The definitive treatment for a localized well-differentiated and low grade dedifferentiated liposarcoma is radical surgical resection or, depending on the tumor size, an excisional biopsy. Optimally, radical resection with negative margins ≥1 cm is possibly curative. Liposarcomas can also be treated with adjuvant radiation and chemotherapy, but this is generally reserved for higher grade tumors. Radiation therapy can be utilized in select cases of low grade, high grade, or superficial tumors >5 cm or low grade deep tumors <5 cm. Adjuvant radiotherapy for high grade lesions does have a benefit in decreasing local recurrence rates from 44% to 26%; however, there does not appear to be an impact on the overall survival rate at 5 years [13]. The side effects of radiation therapy might thus outweigh the benefit since this tumor has a chance of local recurrence but a low potential for metastasis. The overall prognosis is good in a patient with appropriate radical resection. Recurrence rates in high grade dedifferentiated tumors can be as high as 80% and in low grade well-differentiated tumors can range from 5 to 30% depending on anatomical location, clear surgical margins ≥1 cm, and histological type and grade of liposarcoma [13, 14]. Chemotherapy is controversial for localized disease but has demonstrated efficacy with respect to local recurrence, distant recurrence, overall recurrence, and overall survival [15]. However, its benefit has only been observed in higher grade 3 FNCLCC sarcomas [16]. In order to provide optimal care, it is recommended that a patient with a localized liposarcoma be evaluated by a multidisciplinary committee to address consideration for possible surgical resection, radiation, or chemotherapy given consideration for the anatomical site and possible sequelae of therapy versus the pathological aggression of the liposarcoma [7–9].

Since the liposarcoma in this case was mainly a well-differentiated low grade tumor with only a small area of grade 2 dedifferentiation, the risk for metastatic progression is low [17]. The primary risk is local recurrence. The options of possible surgical intervention and radiation were offered to the patient but he decided to undergo surveillance screening. Post operative CT scan demonstrated no recurrence of the liposarcoma. An MRI ordered following the CT scan did not demonstrate any recurrent local tumor. The patient has been followed by surveillance with MRI every six months. It has been 18 months since his liposarcoma resection with no disease recurrence.
